# Encouraging Continuous Usage of Wearable Activity Trackers: The Interplay of Perceived Severity, Susceptibility and Social Media Influencers

**DOI:** 10.3390/ijerph21121549

**Published:** 2024-11-22

**Authors:** Anita Lennox, Re-an Müller, Isaac Sewornu Coffie

**Affiliations:** School of Management Sciences, North-West University, Vanderbijlpark Campus, Vanderbijlpark 1911, South Africa; rean.muller@nwu.ac.za

**Keywords:** wearable activity trackers, smart fitness, consumer attitude, social media influencers, health belief model, cue utilization theory

## Abstract

While past studies have provided enough evidence to show consumer attitude as a key predictor of the adoption and continuous usage intention of wearable activity trackers (WATs), limited studies have examined the antecedents of consumers’ attitudes towards the adoption and continuous usage intention of WATs. Drawing on the health belief model and cue utilization theory, the study seeks to examine the influence of perceived severity and vulnerability as antecedents of consumers’ attitudes towards the adoption and continuous usage intention of WATs as well as the role of social media influencers (SMIs) in influencing continuous usage of WATs. Online survey data from 966 participants (M_age_ = 40.79, STD = 13.49) was analyzed using SPSS 29and AMOS version 29. The result shows that though perceived severity and susceptibility are key significant predictors of consumers’ attitudes towards WATs, the relationship is stronger when SMIs’ personas are used as extrinsic cues. Additionally, while perceived barriers negatively affect consumers’ attitudes towards WATs, the negative effect is neutralized through SMIs’ message framing as an extrinsic cue. Theoretically, the study provides a new insight into the interplay of perceived severity, susceptibility, SMIs’ personas, and message framing on consumers’ attitudes towards the adoption and continuous usage intention of WATs.

## 1. Introduction

Wearable activity trackers (WATs), also known as fitness wearables, are lightweight digital gadgets equipped with internet-connected sensors to track and transmit vital body signals without the involvement of a healthcare practitioner [1,2,3,4,5]. These tracking devices have evolved from simple pedometers to sophisticated smartwatches, wristbands, and rings that allow users to monitor various health metrics such as steps taken, calories burned, heart rate, and sleep patterns. The key objective of a WAT is to help individuals monitor and improve their physical activities and reduce the rate of chronic diseases such as cholesterol-related diseases, cardiovascular diseases, and other noncommunicable chronic diseases, which, according to the World Health Organization (WHO), remain the leading causes of disability and death, taking an estimated 41 million lives each year, equivalent to 74% of all deaths globally [6]. In an era with an increasing prevalence of these lifestyle-related chronic noncommunicable diseases, the promotion of both the adoption and continuous usage of these activity tracking devices is critical to reducing the rate of occurrence of these diseases and achievement of the WHO’s agenda of creating a healthier world by 2030 [7,8,9,10].

Despite their increasing popularity and the health benefits associated with these devices, recent studies and statistics have shown that the rate of adoption and diffusion among consumers is below expectation [11,12]. According to [13], though the global shipment of wearables is forecasted to reach about 560 million units by the end of 2024, there is a 10.5% year-over-year decrease compared to the previous year. Extant research has also shown that consumers’ interest to continue using these devices significantly drops after the initial purchase period with some even discontinuing its usage [14,15,16]. The findings of [16] show that more than half of people who adopt WATs discontinued using them within six months, with less than 20% continuing to use them after that period. [14] observed similar trends in their longitudinal study of Fitbit users, where they found that 25% of participants discontinued use after the first week, and 50% had dropped out by the second week. These statistics and empirical findings call for further investigations to identify factors that will speed up not only the adoption intention but also influence the continuous usage of these devices.

Past studies examining the phenomenon have extensively employed the theory of planned behavior, the technology acceptance model (TAM), self-determination theory, social comparison theory, and unified theory of acceptance and use of technology (UTAUT) to explain factors influencing adoption and usage intention of these wearable activity trackers [2,14,17,18,19,20]. These studies have consistently shown consumer attitude as a key predictor of adoption and continuous usage intention of wearable activity tracking devices, indicating that consumers’ evaluations of these devices play a significant role in their adoption and usage intention. However, while these studies provide valuable insight into the relationship between consumer attitude and intention to adopt and continue using WATs, our examination of the literature shows that there is a dearth of studies that examine the antecedents of consumers’ attitudes towards the adoption and continuous usage intention of WATs, resulting in calls for further research regarding this topic [14,19]. We aim to address this gap by drawing on the health belief model (HBM) to examine the influence of perceived severity, perceived vulnerability, and perceived barriers as antecedents of consumers’ attitudes towards the adoption and continuous usage intention of WATs. According to the HBM, individuals’ evaluations of severity and vulnerability to health conditions (e.g., lifestyle-related chronic diseases) are critical factors that play a significant role in shaping attitudes towards the adoption of recommended health behavior and related products [21,22,23]. Perceived severity refers to an individual’s belief about the seriousness of contracting the health condition. When consumers perceive a health condition as severe and potentially life-altering, they are more likely to take steps to overcome any barrier and develop a favorable attitude towards products that can help them monitor and mitigate the risk of that condition. Similarly, perceived vulnerability, which refers to an individual’s belief about their susceptibility to a health condition, can significantly influence consumer attitudes. Thus, when individuals feel that they are vulnerable or at risk of developing a health condition, they are more likely to adopt preventive measures, including using WATs to avoid or reduce the risk. Though the general effect of these factors has been extensively examined in relation to other health-related issues like vaccine hesitancy [24], infection prevention [21,22,25], breast cancer [26], and climate change [27] in prior studies, scholarly work is scarce on their influence on consumer attitude towards adoption and continuous usage intention of WATs. The study addresses this gap by enhancing our understanding of whether the severity and susceptibility of lifestyle-related health conditions enhance consumers’ evaluations, adoption, and continuous usage intention of WATs. This is particularly significant as past studies have shown that the adoption of WATs, particularly among the youth, is largely influenced by aesthetics/fashion rather than health beliefs [28].

In addition, the study examines the role of social media influencers as an extrinsic cue in shaping consumers’ attitudes towards the adoption and continuous usage intention of WATs. In an increasingly social-media-centric lifestyle era, the use of social media influencers (SMIs) has become a well-known and effective marketing communication tool that marketers use to influence consumers’ attitudes towards brands, capture attention, and shape purchase intentions [28,29,30]. Marketers leverage these influencers to strengthen their marketing efforts and strategies, driving greater awareness, interaction, purchase intention, and loyalty behaviors [29]. For instance, from the source credibility theory’s perspective, past studies have examined and reported the significance of influencers’ endorsement in influencing consumer attitude, purchase intention, eWOM, and brand engagement [30,31]. Although the general effects of influencers on consumer behavior have been examined in prior research [28,31], there is a notable lack of studies investigating the specific impact of the persona and message framing effect (content) of SMIs in shaping consumers’ attitudes towards the adoption and continuous usage intention of WATs (one of the most rapidly growing product categories in the world today). Drawing on the cue utilization theory, the study further fills this gap by examining whether the persona and message framing of SMIs can serve as cues that enhance consumers’ attitudes towards the adoption and continuous usage intention of WATs. According to the cue utilization theory, the way consumers assess products is primarily determined by how effectively marketers align relevant cues with the product and how they communicate these connections [18]. Given that today’s consumers no longer use brands solely for utilitarian purposes but also seek to fulfill self-expression and symbolic needs [28], it is crucial to examine how message framing and the personas of social media influencers can serve as cues that influence consumers’ evaluations of WATs and continued usage intention of these devices.

## 2. Theoretical Foundation

The study is conducted through the lenses of the health belief model (HBM) and the cue utilization theory. The HBM is best suited for this study because it provides six major factors that influence consumers’ decisions to adopt or perform a health-related behavior: perceived severity, perceived susceptibility, perceived benefit, perceived barriers, self-efficacy, and cues to action [22,23]. Perceived severity is an individual’s belief that contracting a health condition will have severe or dangerous implications for their health and finances. On the other hand, perceived susceptibility is the individual’s assessment of how likely they are to experience the health condition. Consumers will only take steps to prevent such conditions when they are convinced after their evaluation that the condition is severe and they are susceptible to the condition [21,23]. Thus, as shown by past studies, though all six variables are necessary to influence health behavior, perceived severity and susceptibility play a major significant role in shaping consumers’ attitudes and adoption behaviors [21,22].

**Figure 1:**
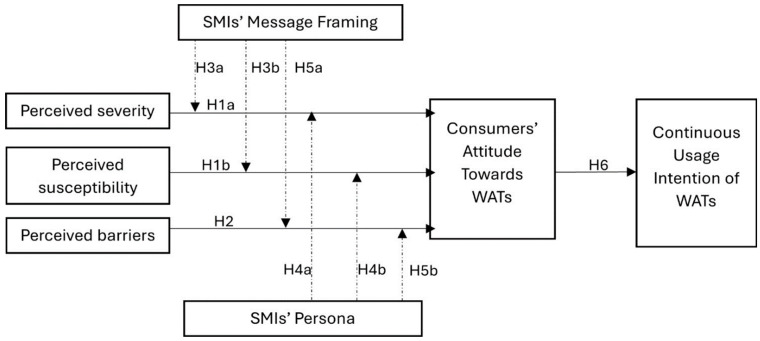
Conceptual framework.

Perceived benefit is the individual’s evaluation of the positive outcomes they will achieve by adopting a health behavior such as using WATs [19]. This is closely linked to perceived barriers, which are the individual’s perceptions of the obstacles that may prevent or make it difficult for them to adopt the health behavior in question [24]. Thus, notwithstanding the benefits, the higher the perceived barriers, the lower the possibility that consumers will engage in preventive measures. Past studies have, for instance, identified data privacy, battery life, and annoyance as major barriers influencing consumers’ intentions to continually use WATs, though they perceived their usage as beneficial (3 Siepmann and Kowalczuk, 2021) [19]. Self-efficacy is one’s confidence in their ability to overcome barriers and successfully perform a behavior they deem beneficial [24]. The higher the self-efficacy, the higher the possibility that a recommended preventive behavior will be adopted and vice versa [32,33]. Cues to action are external or internal triggers that prompt individuals to take action towards adopting a healthy behavior. These cues can significantly influence consumers’ attitudes and adoption intentions, particularly when they align with the individual’s perceived susceptibility and severity of health risk. While prior studies have examined other normative factors like peer influence and social support as significant cues that influence WAT adoption [9,11,34], little attention is given to exploring how social media influencers could influence consumers’ attitudes towards adoption and continuous usage intention of these devices. Additionally, though past studies have extensively examined the effect of perceived benefit/perceived usefulness, perceived barriers, and self-efficacy in other contexts [19,33,35], little scholarly attention has been given to how and whether perceived susceptivity and severity influence consumers’ attitudes towards adoption and continuous usage intention of WATs. We, therefore, generated several hypotheses (see Figure 1).

**H1.**  *(a) Perceived severity and (b) perceived susceptibility of lifestyle-related chronic diseases significantly influence consumer attitudes towards WATs.*

**H2.**  *Perceived barriers are negatively associated with consumers’ attitudes towards WATs.*

### 2.1. The Role of Social Media Influencers

We examine the role of SMIs from the perspective of the cue utilization theory [18]. According to the theory, consumers often rely on cues to draw conclusions about products and use these cues to gather important information to evaluate products [18]. Given its importance in shaping consumers’ perceptions and attitudes about products, strategically associating the right intrinsic and extrinsic cues with products is the key to success [10]. Intrinsic cues are the inherent attributes, qualities, and benefits of a product. Extrinsic cues, on the other hand, are external stimuli like promotional activities, packaging, and other marketing messages [7]. The extant research suggests that extrinsic cues become more influential when intrinsic cues are deficient or insufficient at influencing consumer perception (Woo and Kim, 2021) [10]. For instance, the low long-term usage of WATs suggests that intrinsic cues alone are insufficient to motivate consumers to continue using these devices after their initial adoption and require extrinsic cues to sustain long-term usage intention. As stated earlier, though past studies have examined and found peer pressure, social influence, and aesthetics as significant external cues that propel consumer adoption of WATs, the roles SMIs’ personas and message framing could play have received limited attention.

Strategically, marketers are continually cooperating or partnering with SMIs to influence consumers’ perceptions, attitudes, and purchase intentions of their brands. For example, the strategic cooperation of SMIs and Tom’s of Marine, an oral and personal hygiene company, enabled the company to break through the clutter of greenwashing to achieve a 705% increase in the amount of net new followers and a 28% increase in overall reach with consumers on Instagram who believe in the authenticity and effectiveness of their green products [36]. Leveraging SMIs, Dove was able to alter consumer emotions by championing body positivity and diversity [29]. Influencers go beyond merely providing information in their posts or videos; they play a key role in shaping their audience’s views, perceptions, and attitudes towards a particular product or brand [31]. They play a crucial role in amplifying campaign messages, with their followers often viewing their comments as cues to take direct or indirect actions towards the product in question (Odoom et al., 2024) [29]. Thus, influencer message framing could amplify the severity and susceptibility of lifestyle-related chronic diseases and engender positive attitudes towards WATs.

Beyond their content, the persona of social media influencers, both online and offline, plays a vital role in capturing and engaging their followers’ attention with their messages. An influencer’s persona is the totality of what they stand for and encompasses their personality, values, lifestyle, and the way they present themselves both online and offline [10]. It is carefully crafted to resonate with a specific audience to engender a sense of relatability with followers who do not just like or comment on posts but engage with deep discussion and are willing to act on recommendations [29]. For example, an influencer who consistently promotes a healthy lifestyle, shares personal fitness journeys, and interacts authentically with their followers will likely influence them to value wellness. The consistency of an influencer’s persona makes their followers perceive their messages and endorsements as genuine and view them as someone they would like to emulate [29]. Given that SMIs’ message framing and personas can potentially influence the relationship between perceived severity and susceptibility, as well as consumers’ attitudes towards the adoption and continuous usage of WATs, we hypothesize the following:

**H3.**  *SMIs’ message framing moderates the relationship between (a) perceived severity, (b) perceived susceptibility, and consumers’ attitudes towards WATs such that the relationship is stronger with SMIs’ message framing than without.*

**H4.**  *SMIs’ personas moderate the relationship between (a) perceived severity, (b) perceived susceptibility, and consumers’ attitudes towards WATs such that the relationship is stronger with SMIs’ personas than without.*

**H5.**  *SMIs’ (a) message framing and (b) personas moderate the negative relationship between perceived barriers and consumers’ attitudes towards WATs such that the relationship is stronger with SMIs’ message framing and personas than without.*

### 2.2. Consumer Attitudes Towards WATs and Continuous Usage Intention

According to Ajzen (1991), one of the key determinants or antecedents of individuals’ intention or willingness to engage or perform a recommended behavior is their attitude towards the behavior. Attitude refers to the overall evaluation of a behavior, reflecting an individual’s positive or negative assessment of performing that behavior [21]. In the context of WAT adoption and continuous usage intention, if an individual holds a favorable attitude towards using these devices, they are more likely to develop a strong intention to adopt and consistently use them. The findings of [14] show a positive and significant relationship between consumers’ attitudes and the intention to adopt WATs.

**H6.**  *Consumers’ attitudes towards WATs are positively associated with the continuous usage intention of WATs.*

## 3. Methodology

The hypothesized paths of the study were tested using online data from South African consumers of wearable activity devices who are above 18 years of age. The questionnaires were divided into two major sections. Section A, which is the preliminary section, comprises demographics, WAT usage duration, and eligibility criteria questions. We strictly apply the following eligibility criteria: (a) must be a current user of WATs and (b) following a social media influencer who endorses a fitness activity tracking device. As part of the preliminary section, we provide basic definitions for social media influencers’ message framing and personas, wearable activity trackers, lifestyle-related chronic diseases, susceptibility, and severity. Section B comprises the measurement items where the respondents were asked to indicate their level of agreement or disagreement with these items on a six-point Likert scale. A six-point scale was used because it provides more opportunity for discrimination

The items measuring the various constructs were adapted from prior research but were modified to suit the current context. The items for susceptibility and severity were adapted from Coffie et al. [21,22]. We measured perceived barriers with items from [11,37]. The items we used to measure consumer attitude were from [7,38]. SMIs’ message framing and personas were adapted from [10,28,29,31]. Continued WAT usage intention was measured using items from [7,11,19].

Before we administered the questionnaires, several preliminary steps were taken to enhance the validity of the items. First, the items were given to faculty members (North-West University, Vanderbijlpark Campus, South Africa) with expertise in social media marketing and WATs for vetting for wrong wording and simplicity, after which a pre-test was also undertaken with 37 respondents. For the main data collection, the link for the survey questionnaire was shared on several social media platforms, including Instagram, Facebook, and WhatsApp. Other faculty members were also contacted to help share with their various social media contacts and groups. After twelve weeks of embarking on this exercise, 966 responses were received and subsequently used for data analysis using IBM SPSS 29 and AMOS 29. Of these, 524 were females, 349 were male, and 93 preferred not to disclose their gender. The mean age is 44.57 with a standard deviation of 10.94 (see Table 1).

The study was conducted with ethical approval from the Economic and Management Sciences Research Ethics Committee, North-West University, South Africa (NWU-00899-21-A4).

### Common Method Bias

Since the data for the study were obtained through self-reporting, there was the possibility of common method bias. We, therefore, took several steps (both procedural and statistical) to reduce CMB. In terms of procedure, we assured the respondents of their anonymity, used simple and plain wording to enhance understanding of the items, avoided long items, and worded some items negatively to reduce prediction and socially desirable answers. Reverse coding was, however, conducted for negatively worded items. Harman’s one factor [39] and marker variable approach [40] were conducted on the statistical front. Harman’s single factor, through exploratory factor analysis, shows that out of the 7 factors that explained 68.4% of the variance in the model, the first factor accounted for 24.02% of the variance, an indication that CMB was not an issue. The correlation result between the marker variable (a theoretically unrelated item included in the measurement items) and the dependent variables was low and statistically not significant (r = 0.04, *p* > 0.81) (r = 0.05, *p* > 0.76), also confirming that CMB was not present.

## 4. Results

### 4.1. The Measurement Model

Before testing the proposed hypothesized paths in the study, we examined the measurement items’ validity and reliability. We assessed the reliability of the items by following the recommended thresholds of the standardized factor loadings of the various items: Cronbach’s alpha (α), composite reliability (CR), and average variance extracted (AVE) [41]. The results (see Table 2) show that the factor loadings of all the items exceeded the 0.6 thresholds, the 0.7 thresholds for α and CR, and the 0.5 thresholds for AVE. Discriminant validity is also achieved (see Table 3) as the square root of the AVEs (diagonal values) is higher than the corresponding correlations (off-diagonal values) [42].

We further examined the fitness of the hypothesized model through confirmatory factor analysis by applying the maximum likelihood estimation method with robust standard errors to evaluate the entire model. The fitness of the hypothesized model was deemed fit as all the fit indices were above the recommended thresholds (χ^2^ = 977.307, df = 419, χ^2^/df = 2.333, CFI = 0.949, NFI = 0.916, GFI = 0.889, TLI = 0.949, RMSEA = 0.053, SRMR = 0.058) (see Table 2). Specifically, χ^2^/df < 0.3, CFI < 0.9, NFI > 0.9, GFI > 0.9, TLI > 0.9, RMSEA < 0.08, and SRMR < 0.08 [43]. A ratio of χ^2^/df = 2.333 is typically interpreted as acceptable when below 3, suggesting a reasonable fit of the model to the data. CFI is a relatively robust fit index, with values above 0.90 considered acceptable and above 0.95 indicative of an excellent fit. Thus, the reported CFI of 0.949 indicates that the model fits the data very well. NFI assesses model fit in comparison to a baseline model, with values exceeding 0.90 denoting an acceptable fit. The NFI of 0.916 signifies that the current model is adequate. GFI measures how well the model explains the observed covariance matrix. Generally, a GFI above 0.90 is preferred, and although 0.889 is slightly below this threshold, it is still within an acceptable range. TLI evaluates model fit with respect to the data, with values over 0.95 indicating a very good fit. The TLI of 0.949 suggests a good fit of the model. RMSEA is an index where lower values are preferred. Generally, a value below 0.06 is acceptable, and below 0.05 indicates a very good fit. The RMSEA of 0.053 reflects a fitting model. SRMR represents the standardized average difference between observed and predicted values, with values below 0.08 indicating acceptable fit. The SRMR of 0.058 is within the acceptable range, suggesting good model fit [43]. Thus, the model demonstrates strong fit across several indicators, with most metrics falling within acceptable ranges, supporting the conclusion that the model fits the data well.

The standardized factor loading quantifies the strength of the relationship between observed variables and latent variables. Values above 0.50 are typically considered acceptable, while values exceeding 0.70 indicate a strong relationship. High SFL values suggest that the observed variable adequately measures the latent construct, while lower values may imply a less accurate reflection.

### 4.2. The Assessment of the Structural Model

**Figure 2:**
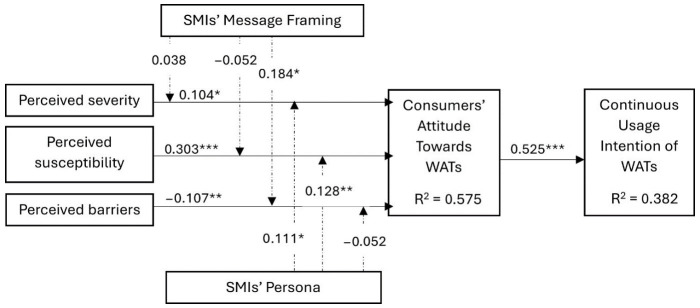
Structural model. Note: * = *p* < 0.05; ** = *p* < 0.01; *** = *p* < 0.001.

After determining the model’s reliability, validity, and fitness, we tested the hypothesized paths using structural equation modeling with AMOS version 29. The model explained 57.5% and 38.2% of the variance in consumers’ attitudes and continuous usage intention of WATs, respectively (Figure 2). The structural model’s goodness-of-fit indices include χ^2^ = 324.630, df = 149, χ^2^/df = 2.179, CFI = 0.920, NFI = 0.887, TLI = 0.913, TLI = 0.922, RMSEA = 0.042, and SRMR = 0.041. The analysis of the directly hypothesized paths (see Table 4) shows that perceived severity (β = 0.104, *p* < 0.05) and perceived susceptibility (β = 0.302, *p* < 0.01) has a significant positive direct effect on consumers’ attitudes towards wearable activity trackers, providing support for H1a and H1b. Perceived barriers were also found to have a negative significant influence on consumers’ attitudes towards WATs (β = −0.107, *p* < 0.05). The result also provides support for H6, suggesting that consumers’ attitudes are positively associated with continuous usage intention of WATs (β = 0.525, *p* < 0.01).

### 4.3. The Moderation Result

We followed the approach of [44] to test the interactive effect of SMIs’ message framing (content) and SMIs’ personas on the relationships between consumers’ perceptions of susceptibility and the severity of lifestyle-related chronic diseases and attitudes towards WATs by mean-centering the constructs used for the interaction terms. This approach is suitable for dealing with multicollinearity and endogeneity concerns, especially when both predictor and outcome variables are measured on continuous scales [44]. The result shows that (see Table 5) influencers’ message framing has no significant interactive effect on both perceived severity (β = 0.038, *p* > 0.05) and susceptibility (β = −0.052, *p* > 0.05), thereby providing no support to H3a and H3b, but it does have significant positive interactive effects on the negative relationship between perceived barriers and consumers’ attitudes towards WATs (β = 0.184, *p* < 0.05). Regarding the interactive effect of SMIs’ personas, the result reveals that while SMIs’ personas significantly moderate the relationships between perceived susceptibility (β = 0.128, *p* < 0.05) and severity (β= 0.111, *p* < 0.05) and consumers’ attitudes towards WATs, it has no significant interactive effect on perceived barriers and consumers’ attitudes towards WATs (β = −0.052, *p* >0.05).

## 5. Discussion

The primary objective of this study is to examine the influence of perceived susceptibility and severity of health conditions like lifestyle/health-related diseases as key antecedents of consumers’ attitudes towards the adoption and continuous usage intention of WATs as well as the role of SMIs in influencing the perception of severity and susceptibility in shaping consumers’ attitudes towards the adoption and continuous usage of these devices. Specifically, we examined (a) the direct relationship between perceived susceptibility and severity of lifestyle/health-related diseases on consumers’ attitudes towards WATs, (b) the interactive influence of SMIs’ personas and message framing on the direct relationship between susceptibility, severity, and consumers’ attitudes towards WATs, and (c) the role of SMIs in mitigating the effect of perceived barriers on consumers’ attitudes towards WATs.

Like most prior studies (e.g., [9,11], the current study confirms that consumers’ attitudes towards WATs have a positive significant influence on the continuous usage intention of WATs. Thus, the study again shows that consumers’ evaluations of WATs play a key role in consumers’ intention to continue to use these devices.

The study expands on the current literature and provides evidence to show that consumers’ perceptions of the severity and susceptibility of a health condition like lifestyle-related diseases are key antecedents or predictors of consumers’ attitudes towards WATs. The result specifically shows that perceived susceptibility and severity have a significant positive influence on consumers’ attitudes towards WATs, suggesting that these variables play a significant role in shaping consumers’ attitudes towards WATs and, by extension, their intention as to whether to continue their usage, not just the perceived benefit/usefulness or aesthetics. This agrees with the position of [22,45] that perception of severity and susceptibility plays an important role in shaping individuals’ attitudes towards health-related behaviors and products.

The results further provide evidence to show that perceived barriers significantly negatively influence consumers’ attitudes towards WATs. This indicates that consumers’ experiences or perceptions of barriers will significantly influence their negative evaluations and attitudes towards adoption and continuous usage intention. Thus, reducing perceived barriers is crucial to improving consumers’ attitudes and continuous usage intention of WATs. The current result aligns with past findings [19,37].

Regarding the interactive role of social media influencers’ personas and message framing, the result shows that the persona of SMIs significantly moderates the positive relationship between perceived susceptibility and severity and consumers’ attitudes towards WATs. This confirms our hypotheses that the persona of an SMI as an extrinsic cue will strengthen or ameliorate consumers’ perceptions of the severity and vulnerability of health conditions like lifestyle-related diseases and positive evaluation of WATs. The result further shows that SMIs’ message framing has a significant positive interactive effect on the negative relationship between perceived barriers and consumers’ attitudes towards WATs, suggesting that the negative influence of perceived barriers on consumers’ attitudes towards WATs is significantly mitigated or reduced by the content or message framing of SMIs. Thus, despite acknowledgeable barriers or challenges associated with WATs, SMI message framing could significantly reduce consumers’ negative attitudes and improve the continuous usage intention of these devices. SMIs’ message framing only moderated the relationship between perceived barriers and consumers’ attitudes but had no moderating effect on the relationships between susceptibility, severity, and consumers’ attitudes. Likewise, while persona moderated the relationships between susceptibility, severity, and consumers’ attitudes, it did not moderate the relationship between perceived barriers and attitudes towards WATs. The current finding disagrees with the findings of [10] that message framing has no significant influence on consumers’ evaluations of WATs. Our findings show that when message framing to promote these devices is achieved through SMIs, it significantly influences their evaluation of barriers to adoption and continuous usage intention of these wearable devices. The result validates the findings of [29] that message framing by SMIs reduces consumers’ negative perceptions, attitudes, and hate for a brand.

## 6. Theoretical Contribution

While WATs are technological products with the core objective of enhancing the performance of health-related behaviors among individuals, past studies have largely examined their adoption from technological perspectives, with limited attention given to using health-related theories to examine factors influencing their adoption and continuous usage intention. We addressed this gap and contributed to the literature by using the health belief model to examine factors influencing consumers’ attitudes towards the adoption and continuous usage intention of these devices, with the result providing evidence to show perceived susceptibility and severity as key antecedents of consumers’ positive evaluation of WATs, which are critical for the continuous usage intention of these devices. The study expands the HBM beyond traditional health communication settings to a contemporary marketing issue, demonstrating its relevance in understanding consumer behavior in a digital human-to-computer environment.

As one of the fastest-growing product categories, the WAT has received much academic interest in the last decade. The major concern has, however, shifted from factors influencing its adoption to understanding drivers and barriers of its continuous usage, an aspect which is still in its early stages and requires research. We contribute to the literature by focusing on factors influencing consumers’ attitudes and the intention to use these wearable devices continuously. Though past studies have identified barriers such as device annoyance, loss of tracking motivation, privacy, and security concerns, limited studies have examined how these barriers could be reduced or mitigated. We contribute to the literature by empirically showing that message framing by social media influencers as an extrinsic cue could significantly neutralize or mitigate the negative influence of these barriers on consumers’ evaluations and attitudes towards adoption and continuous usage of WATs.

Additionally, we contribute to the existing literature by empirically showing influencers’ personas as important extrinsic cues that significantly strengthen consumers’ perceptions of severity and susceptibility to health conditions like lifestyle-related diseases, thereby increasing their positive evaluation of WATs and the continuous usage intention among consumers. The study further contributes to the WAT literature by expanding on the current conceptualization of how consumer attitudes towards WATs are formed by showing that attitudes are not only formed by intrinsic health beliefs but also by extrinsic marketing cues such as influencer persona and message framing. With this perspective, the study shows that consumer attitudes towards health technologies are more impressionable and can be influenced by strategically selected external factors, providing a more dynamic understanding of consumer attitude formation towards health-related products and behavior.

Finally, the study makes a novel contribution by integrating the HBM with the cue utilization theory to examine the antecedents of consumer attitudes and continuous usage intentions of WATs. The theoretical integration of these two theories provides a more holistic understanding of how health-related perceptions (like perceived susceptibility and severity) interact with external cues (like SMI personas and message framing) to shape consumer behavior.

### 6.1. Practical Implications

Drawing on the result of the study, we provide the following recommendations for managers and policy. First, since the result shows that perceived barriers like privacy and security concerns have a significant negative effect on consumers’ attitudes towards WATs and their continuous usage intention, we recommend that the government and policymakers get involved and enact clear data protection policies on how personal information could be acquired and used by third parties. This is important because WATs have become an important aspect of health management, and patient privacy regulations should be extended to their usage. Based on the results, using SMI message framing to communicate these policies could play a significant role in how consumers assimilate these policies and thereby help to reduce the negative consumer attitudes towards WATs and their continuous usage. Since health insurance funds are one of the biggest beneficiaries of a healthy population, we recommend that they support advocating for these data protection policies regarding the use of these devices and could also support by engaging influencers to use their message framing and personas to shape consumer attitudes and continuous usage intention of WATs.

Second, our result shows that perceived severity and susceptibility to health conditions like lifestyle-related chronic diseases have a significant positive influence on consumers’ attitudes towards WATs and their intention to use them continuously. Based on this result, we recommend that marketers highlight or emphasize the severity of these lifestyle-related diseases and how increasingly consumers have become vulnerable to these conditions, largely due to lifestyles like sitting for long hours. As shown by our findings, doing this with strategically selected SMIs with the right personas will yield or have a stronger impact on consumers’ positive evaluations and attitudes towards the continuous usage intention of WATs.

### 6.2. Limitations and Future Research Directions

Despite the contributions, the study has some limitations that need to be addressed by future research. The current study is a cross-sectional survey, which does not allow causal relationships between variables to be tested. To gain a deeper understanding of the phenomenon, longitudinal studies would be appropriate. Different age groups respond to the same stimuli differently. The study does not examine how different age groups perceived susceptibility and severity of lifestyle-related chronic disease and their attitude towards WATs and continuous usage of these devices. Addressing this in future research and how SMI characteristics influence this perception and attitude towards continuous usage intention of WATs will be an important contribution to knowledge. We examined SMIs’ persona and message framing as external cues that play a significant role in shaping consumer attitudes towards the continuous usage intention of WATs. Future research could examine how different types of SMI personas (e.g., health professionals vs. lifestyle influencers) could affect the relationship between health beliefs and consumer attitudes towards WATs and their continuous usage intention. Again, though cultural differences might have a significant influence on consumers’ attitudes towards WATs and SMIs, the current study does not consider the effect of cultural differences on these relationships. Future research should empirically test this effect to enhance practical understanding of how to promote continuous usage of these devices in different cultural settings. Since factors such as socioeconomic status, health consciousness, or previous health conditions may confound the relationship between perceived severity and WAT usage, we recommend further examination of these factors in future research. Finally, different types of WATs (e.g., basic pedometers vs. advanced smartwatches) may have varying appeal and usage rates. Future research could explore the interactive effects of device type to provide more nuanced consumer attitudes towards continuous usage of these devices.

## Figures and Tables

**Table 1:** Demographic profile of respondents.

	Freq.	%
Gender		
Male	524	54.2
Female	349	36.2
Non-binary	-	-
Prefer not to disclose	93	9.6
Age		
18–26	98	10.1
27–36	121	12.5
37–46	210	21.7
47–56	465	48.1
Above 57	72	7.5
Income (USD)		
<1000	588	60.9
1000–2000	339	35.1
2001–3000	30	3.1
3001–4000	7	7
4001–5000	2	2
Above 5000	-	-

**Table 2:** Measurement model.

Measurement Items	SFL
Perceived severity (α = 0.891; CR = 0.892; AVE = 0.674)	
Chronic diseases are dangerous to get	0.847
I am afraid to even think of getting a chronic disease	0.829
My financial security would be in danger if I get chronic disease	0.814
When I see people with chronic diseases, I feel terrified	0.792
Perceived susceptibility (α = 0.854; CR = 0.861; AVE = 0.609)	
My chances of getting chronic disease is higher if I don’t monitor my health	0.758
Although I am healthy now, if I don’t monitor my physical activity, I will probably get chronic disease in the future	0.847
I will most likely get chronic disease if I don’t check and improve my health lifestyle	0.838
* I am not worried if I stop monitoring my fitness	0.666
Perceived barriers (α = 0.890; CR = 0.890; AVE = 0.670)	
* I am not concerned that a fitness wearable collects too much personal information	0.840
I am concerned that fitness wearable providers might use my personal information	0.808
Disturbs me with exercise	0.858
Hackers could access my personal information when I use fitness wearables	0.766
SMI message framing (α = 0.911; CR = 0.911; AVE = 0.720)	
Post that demonstrates expertise and makes tracking more convincing	0.835
The messages resonate with my health concerns	0.849
Addresses my concerns about tracking devices	0.861
I like contents with visually appealing aesthetics	0.849
SMI persona (α = 0.900; CR = 0.902; AVE = 0.648)	
* I don’t like influencers with credible opinions on health issues	0.797
I follow influencers who are physically attractive	0.849
I like influencers who always wear activity trackers	0.836
I feel a personal connection with health-conscious influencers	0.781
My perceptions often change when I receive information from the influencers whom I follow	0.757
Consumer attitudes (α = 0.937; CR = 0.938; AVE = 0.716)	
Using a wearable activity tracking device is a good thing	0.873
* I don’t like the idea of using an activity tracking device to monitor my health	0.867
Activity tracking devices are comfortable to wear	0.877
My activity tracker makes me be more conscious of my health	0.860
Using my activity tracking device motivates me to work towards a healthier lifestyle.	0.817
Using my activity tracker keeps me motivated to live healthier	0.779
Continuous usage intention (α = 0.856; CR = 0.857; AVE = 0.600)	
I intend to continue using my activity tracker rather than stopping using it	0.792
I plan to continue using my activity tracking device	0.719
I predict I would wear an activity tracking device in the next several years	0.815
I intend to make wearing an activity tracking device a habit	0.77
χ^2^ = 977.307, df = 419, χ^2^/df = 2.333, CFI = 0.949, NFI = 0.916, GFI = 0.889, TLI = 0.949, RMSEA = 0.053, SRMR = 0.058	

Note: * these items were reversed coded.

**Table 3:** Discriminant validity.

	Mean	STD	1	2	3	4	5	6	7
Perceived Severity	5.017	0.935	***0.821***						
Perceived Susceptibility	4.238	1.174	0.222 **	***0.781***					
SMI Message Framing	4.914	0.858	0.510 **	0.469 **	***0.849***				
SMI Persona	4.925	0.920	0.276 **	0.372 **	0.478 **	***0.805***			
Perceived Barriers	4.638	1.010	0.296 **	0.411 **	0.528 **	0.481 **	***0.819***		
Consumer Attitude	4.619	1.095	0.440 **	0.448 **	0.326 **	0.311 **	0.494 **	***0.846***	
Continuous Usage Intention	4.864	0.930	0.485 **	0.472 **	0.432 **	0.459 **	0.374 **	0.318 **	***0.775***

** significant in two-tailed test. The bold and the italics are the square root of the AVEs.

**Table 4:** Direct paths.

	Hypothesized Paths			B	S.E	C.R	P	Decision
H1a	Attitude	<---	P_Severity	0.104	0.044	2.368	0.018	Supported
H1b	Attitude	<---	P_Susceptibility	0.302	0.034	8.987	***	Supported
H2	Attitude	<---	Barriers	−0.107	0.040	−2.660	0.008	Supported
H6	Intention	<---	Attitude	0.525	0.030	17.332	***	Supported

Note: *** = *p* < 0.001.

**Table 5:** Moderation analysis.

	Hypothesized Interactive Path			B	S.E	C.R	P	Decision
H3a	Attitude	<---	SMI Message Framing × Severity	0.038	0.059	0.654	0.513	Not supported
H3b	Attitude	<---	SMI Message Framing × Susceptibility	−0.052	0.047	−1.101	0.271	Not supported
H4a	Attitude	<---	SMI Persona × Severity	0.111	0.050	2.229	0.026	Supported
H4b	Attitude	<---	SMI Persona × Susceptibility	0.128	0.044	2.881	0.004	Supported
H5a	Attitude	<---	SMI Message framing × Barriers	0.184	0.074	2.503	0.012	Supported
H5b	Attitude	<---	SMI Persona × Barriers	−0.052	0.046	−1.114	0.265	Not supported

## Data Availability

Data is available upon request from the corresponding author.
